# Structure and Sequence of the Human Fast Skeletal Troponin T (TNNT3) Gene: Insight Into the Evolution of the Gene and the Origin of the Developmentally Regulated Isoforms

**DOI:** 10.1002/cfg.343

**Published:** 2003-12

**Authors:** Raymund Stefancsik, Jeffrey D. Randall, Chengjian Mao, Satyapriya Sarkar

**Affiliations:** 1 Department of Anatomy and Cellular Biology Tufts University Health Science Campus 136 Harrison Avenue, M&V 519 Boston MA 02111 USA; 2 Graduate Program in Cell Molecular and Developmental Biology Sackler School of Biomedical Sciences Tufts University Boston MA 02111 USA; 3 Department of Biomedical Sciences Tufts University School of Veterinary Medicine Boston MA 02111 USA; 4 Veterinary Medical Research Institute of the Hungarian Academy of Sciences Hungaria krt 21 Budapest H-1143 Hungary; 5 U.S. Genomics Woburn MA 0181 USA; 6 Department of Biochemistry University of Illinois at Urbana-Champaign Urbana IL USA

## Abstract

We describe the cloning, sequencing and structure of the human fast skeletal troponin T (TNNT3) gene located on chromosome 11p15.5. The single-copy gene encodes 19 exons and 18 introns. Eleven of these exons, 1–3, 9–15 and 18, are constitutively spliced, whereas exons 4–8 are alternatively spliced. The gene contains an additional subset of developmentally regulated and alternatively spliced exons, including a foetal exon located between exon 8 and 9 and exon 16 or α (adult) and 17 or β (foetal and neonatal). Exon phasing suggests that the majority of the alternatively spliced exons located at the 5′ end of the gene may have evolved as a result of exon shuffling, because they are of the same phase class. In contrast, the 3′ exons encoding an evolutionarily conserved heptad repeat domain, shared by both TnT and troponin I (TnI), may be remnants of an ancient ancestral gene. The sequence of the 5′ flanking region shows that the putative promoter contains motifs including binding sites for MyoD, MEF-2 and several transcription factors which may play a role in transcriptional regulation and tissue-specific expression of TnT. The coding region of TNNT3 exhibits strong similarity to the corresponding rat sequence. However, unlike the rat TnT gene, TNNT3 possesses two repeat regions of CCA and TC. The exclusive presence of these repetitive elements in the human gene indicates divergence in the evolutionary dynamics of mammalian TnT genes. Homologous muscle-specific splicing enhancer motifs are present in the introns upstream and downstream of the foetal exon, and may play a role in the developmental pattern of alternative splicing of the gene. The genomic correlates of TNNT3 are relevant to our understanding of the evolution and regulation of expression of the gene, as well as the structure and function of the protein isoforms. The nucleotide sequence of TNNT3 has been submitted to EMBL/GenBank under Accession No. AF026276.


**Comparative and Functional Genomics**
*Comp Funct Genom* 2003; **4**: 609–625.
Published online in Wiley InterScience (www.interscience.wiley.com). **DOI:** 10.1002/cfg.343

**Primary Research Paper**

### Structure and sequence of the human fast skeletal troponin T (TNNT3) gene: insight into the evolution of the gene and the origin of the developmentally regulated isoforms

Raymund Stefancsik[1,2#], Jeffrey D. Randall[1,2##], Chengjian Mao[1,###] and Satyapriya Sarkar[1,2,3*]

[1] *Department of Anatomy and Cellular Biology, Tufts University, Boston, MA 02111, USA*
[2] *Graduate Program in Cell, Molecular and Developmental Biology, Sackler School of Biomedical Sciences, Tufts University, Boston, MA 02111, USA*
[3] *Department of Biomedical Sciences, Tufts University School of Veterinary Medicine; Boston, MA 02111, USA*

**Correspondence to: Satyapriya Sarkar, Department of Anatomy and Cellular Biology, Tufts University, Health Science Campus, 136 Harrison Avenue, M&V 519, Boston, MA 02111, USA. E-mail: satyapriya.sarkar@tufts.edu*

*# Present address: Veterinary Medical Research Institute of the Hungarian Academy of Sciences, H-1143 Budapest, Hungaria krt 21, Hungary.*

*## Present address: U.S. Genomics, Woburn, MA 0181, USA.*

*### Present address: Department of Biochemistry, University of Illinois at Urbana-Champaign, Urbana, IL, USA.*

### Abstract

**We describe the cloning, sequencing and structure of the human fast skeletal troponin T (TNNT3) gene located on chromosome 11p15.5. The single-copy gene encodes 19 exons and 18 introns. Eleven of these exons, 1–3, 9–15 and 18, are constitutively spliced, whereas exons 4–8 are alternatively spliced. The gene contains an additional subset of developmentally regulated and alternatively spliced exons, including a foetal exon located between exon 8 and 9 and exon 16 or $\alpha$ (adult) and 17 or $\beta$ (foetal and neonatal). Exon phasing suggests that the majority of the alternatively spliced exons located at the 5′ end of the gene may have evolved as a result of exon shuffling, because they are of the same phase class. In contrast, the 3′ exons encoding an evolutionarily conserved heptad repeat domain, shared by both TnT and troponin I (TnI), may be remnants of an ancient ancestral gene. The sequence of the 5′ flanking region shows that the putative promoter contains motifs including binding sites for MyoD, MEF-2 and several transcription factors which may play a role in transcriptional regulation and tissue-specific expression of TnT. The coding region of TNNT3 exhibits strong similarity to the corresponding rat sequence. However, unlike the rat TnT gene, TNNT3 possesses two repeat regions of CCA and TC. The exclusive presence of these repetitive elements in the human gene indicates divergence in the evolutionary dynamics of mammalian TnT genes. Homologous muscle-specific splicing enhancer motifs are present in the introns upstream and downstream of the foetal exon, and may play a role in the developmental pattern of alternative splicing of the gene. The genomic correlates of TNNT3 are relevant to our understanding of the evolution and regulation of expression of the gene, as well as the structure and function of the protein isoforms. The nucleotide sequence of TNNT3 has been submitted to EMBL/GenBank under Accession No. AF026276. Copyright © 2003 John Wiley & Sons, Ltd.**

Received: 18 July 2003
Revised: 24 September 2003
Accepted: 6 October 2003

**Keywords: human fast skeletal troponin T (TNNT3) gene; structure and sequence of TNNT3; promoter and *cis* elements of TNNT3; alternative and constitutive splicing of exons; foetal exon; evolution of TNNT3 gene; muscle-specific splicing enhancer; exon shuffling**

### Introduction

The regulation of vertebrate striated muscle contraction by $Ca^{2+}$ is mediated through the $Ca^{2+}$ regulatory tropomyosin (Tm)–troponin (Tn), a myofibrillar protein complex (Tm–Tn) located in thin filaments (reviewed in Gordon *et al.*, 2000). The Tm–Tn complex binds to filamentous or

Copyright © 2003 John Wiley & Sons, Ltd.

polymerized actin (F-actin) to form the regulated thin filament. The Tn complex consists of three structurally and functionally different subunits: troponin C (TnC), troponin I (TnI) and troponin T (TnT). TnC binds $Ca^{2+}$, TnI binds to actin and prevents muscle contraction by inhibiting actin–myosin interaction, whereas TnT attaches the Tn complex to Tm. The binding of $Ca^{2+}$ to TnC triggers muscle contraction through a process of 'information transfer' that is propagated by protein–protein interactions and conformational changes in virtually all thin filament proteins. All of these proteins play specific roles in, and are therefore required for, reversible $Ca^{2+}$-dependent regulation of contraction. The final consequence is the alteration of a kinetic step in the myosin ATPase reaction scheme.

TnT is not only a structural link between the Tn complex and Tm, but is also essential for $Ca^{2+}$ sensitivity in striated muscle (Farah and Reinach, 1995; Perry, 1998). TnT increases the cooperativity of actin–tropomyosin binding to myosin. In addition to the *in vitro* biochemical studies, genetic evidence also supports the importance of TnT in the regulation of muscle contraction. For example, mutants of a TnT homologue (*mup-2*) in *Caenorhabditis elegans* are defective for embryonic body wall muscle cell contraction, sarcomere organization and cell positioning (Myers *et al.*, 1996), most probably due to hypercontraction and delayed muscle relaxation (McArdle *et al.*, 1998). Mutations in the *Drosophila melanogaster* TnT gene (*upheld* and *indented thorax*) result in myofibrillar abnormalities in flight muscles (Fyrberg *et al.*, 1990). A certain form of familial hypertrophic cardiomyopathy, which is an inherited human disease, is caused by cardiac TnT mutations (Thierfelder *et al.*, 1994; Watkins *et al.*, 1995).

In vertebrates, the proteins of the Tn complex are encoded by separate multigene families whose members are expressed in a muscle fibre type-specific manner. Accordingly, there are three vertebrate TnT isoform genes: slow skeletal, fast skeletal and cardiac. The three human TnT genes are located on different chromosomes in the genome. The human slow skeletal TnT (TNNT1) and the human cardiac TnT (TNNT2) isoform genes have been assigned to chromosomes 19q13.4 and 1q32, respectively (Mesnard *et al*, 1995; Samson *et al.*, 1992). The human fast skeletal TnT (TNNT3) gene has been mapped to 11p15.5 (Mao *et al.*, 1996).

The locus 11p15.5 harbours several genes that are imprinted depending on the parental origin. These imprinted genes include H19, IGF2 (insulin-like growth factor II), INS (insulin), ASCL2 (achaete-scute homologue 2), CDKN1C ($P57^{KIP2}$), IPL and IMPT1 clustered in this region of chromosome 11 (Barlow, 1995; Dao *et al.*, 1998; Matsuoka *et al.*, 1996; Miyamoto *et al.*, 1996; Qian *et al.*, 1997). Due to the presence of genetically imprinted genes in 11p15.5, this chromosomal region has been intensively studied in human genomic research. Although the TNNT3 gene shows biallelical embryonic expression (Yuan *et al.*, 1996), the comparison of the nucleotide sequence of the imprinted genes in this locus with that of TNNT3 may provide important clues regarding the *cis* signals and mechanisms for parent-of-origin specific regional imprinting on the short arm of chromosome 11. For example, parallel expression of H19 and TNNT3 in different adult skeletal muscle types suggests that these genes may share an enhancer (Yuan *et al.*, 1996).

The sub-localization of TNNT3 to band p15.5 on chromosome 11 (Mao *et al.*, 1996) may have clinical relevance. A clinical disorder, Beckwith–Wiedemann syndrome (BWS) as well as various childhood and adult tumour-related abnormalities including rhabdomyosarcoma, have been mapped to this locus (Pettenati *et al.*, 1986; Henry *et al.*, 1993). The possible involvement of genomic correlates of TNNT3 in muscular hypertrophy associated with BWS remains to be studied.

In contrast to TnC and TnI, which do not show any alternatively spliced isoform, vertebrate fast skeletal TnT is a paradigm for studying complex exon-splicing patterns from a single gene (Breitbart *et al.*, 1985). Developmentally regulated splicing of a new exon in the fast troponin T gene has been described in foetal and neonatal rabbit and rat muscle (Briggs and Schachat, 1993; Morgan *et al.*, 1993). It has been designated 'f', for foetal exon (Briggs and Schachat, 1993). An 'f' exon homologue has also been proposed to be present (Briggs *et al.*, 1994) in the human fast skeletal TnT $\beta$ isoform cDNA reported by us (Wu *et al.*, 1994). By determining the complete exon–intron organization of the human fast skeletal TnT gene, we present conclusive evidence for the existence of a 'foetal' exon in TNNT3 together with some information on the potential *cis* elements regulating its splicing during development.

Copyright © 2003 John Wiley & Sons, Ltd. 

Comparative sequence analysis of human $\beta$ TnT with other vertebrate striated TnT isoforms, previously carried out by us (Wu *et al.*, 1994) has shown that the TnT isoforms have a conserved central region flanked by the variable carboxy-terminal and an extremely variable amino-terminal segment. The Tm binding site and the interaction site with TnI are important segments of this conserved region in TnT isoforms. With the availability of new TnT sequences, particularly from invertebrate species, extensive computer-assisted analysis of phylogenetically distant TnT amino acid sequences carried out by us identified a highly conserved protein domain that is characterized by a seven amino acid heptad repeat units (HR) motif with a potential for $\alpha$-helical coiled-coil formation (Stefancsik *et al.*, 1998). This conserved domain, spanning over 60–70 amino acid residues, is present in TnT sequences from mammals, birds, *Caenorhabditis elegans, Drosophila* and the protochordate ascidian *Halocynthia roretzi*. A similar HR domain is also conserved in all known TnI sequences. Interestingly, the conserved HR domains of TnI and TnT show similarity at a statistically significant level, suggesting that the HR domains of these two polypeptides may have a common ancestry. Furthermore, it was observed that the conserved HR domains are primarily involved in TnI–TnT dimerization, presumably through the formation of $\alpha$-helical coiled coils (Stefancsik *et al.*, 1998). It is also of interest to investigate the molecular evolution of the conserved HR domain of TnT at the genomic level. Information on the exon–intron structure of TNNT3 can facilitate these studies.

In the present study we describe the characterization and sequencing of TNNT3. Comparative studies with genomic and cDNA sequences available in the public databases reveal interesting implications for the regulation and evolution of this important sarcomeric protein gene.

### Materials and methods

### Isolation of genomic fragments of TNNT3 representing the protein coding exons

Using information from the cDNA sequence of the $\beta$ embryonic isoform of human TNNT3 (Wu *et al.*, 1994) and the exon and intron organization of the rat TNNT3 gene (Breitbart and Nadal-Ginard, 1986), two pairs of primers were designed:

- P24 (5′-caccttcaccatgtctgacgaggaa-3′), which locates at the boundary of the 5′ untranslated region and ATG start codon; and
- P6′ (5′-ggcttcaaagtggctgtcgatg-3′), which is located in the middle in the cDNA.
- P19 (5′-ctcatcgacagccactttgaag-3′) is located in exon 11 of the cDNA; and
- P4 (5′-gagacccctgacaggattgtgg-3′) is located in the 3′ untranslated region.

The pair P24 and P6′ was used to amplify a genomic fragment of about 12 kb; the pair P19 and P4 was used to amplify a fragment of about 5 kb. The long polymerase chain reaction (PCR) for the 12 and 5 kb fragments was carried out according to the manufacturer's recommendations (Boehringer-Mannheim). The amplified fragments obtained by using these two pairs of primers overlap and include the protein-coding region.

### Subcloning of the PCR-generated DNA fragments

The PCR products were purified from low melting point agarose gel (Sambrook *et al.*, 1989). The 5 kb fragment was cloned into pBluescript II SK (Stratagene) at the *Sma*I site, after T4 polymerase treatment to generate a blunt-ended DNA fragment and phosphorylation by polynucleotide kinase. Considering the large size of the 12 kb fragment and the inefficiency of cloning large DNA fragments into pBluescript II SK, we chose an alternative approach to subcloning the 12 kb fragment. After T4 polymerase and polynucleotide kinase reactions, the 12 kb fragment was digested with *Hinc*II. This produced five smaller fragments with blunt ends. These fragments were resolved and purified from a low melting point agarose/EtBr gel and subcloned into pBluescript individually. Fragments were sequenced using standard methods. Using information from the sequence, primer pairs were designed to amplify by PCR the flanking regions of *Hinc*II sites to test the possibility that some fragments may be lost because of their small size. Similarly these fragments were subcloned into pBluescript and sequenced. The resulting PCR amplified fragments cover all the boundary regions between each of the adjoining five main fragments.

Copyright © 2003 John Wiley & Sons, Ltd. 

### PCR amplification, cloning and sequencing of intron 1 of TNNT3

Since the published nucleotide sequence of the human $\beta$TnT cDNA (Wu *et al.*, 1994) does not extend to the first exon, we searched the human expressed sequence tag (EST) database of GenBank (**http://www.ncbi.nlm.nih.gov**) using the 'Gapped BLAST' program for potential sequences containing this region (Altschul *et al.*, 1997). We indeed found a partial human cDNA sequence (Accession No. AA194223) that includes a major part of exon 1. After aligning this sequence with the rat and mouse homologues (Figure 1), we were able to deduce the boundary of exons 1 and 2.

Accordingly, we designed a sense primer corresponding to the 3′ end of exon 1 (IN11s; 5′-ccttctcacactcgacccgcag-3′) and an antisense primer (IN12a; 5′-acttcctcgtcagacatggtg-3′) corresponding to the 5′ end of exon 2. By using the IN11s–IN12a primer pair with the long PCR kit of Boehringer-Mannheim, a 3.1 kb genomic DNA fragment was amplified. The PCR template was human genomic DNA purchased from Boehringer-Mannheim. The 3.1 kb fragment was subcloned using the pCR® 2.1-TOPO cloning kit from Invitrogen (Carlsbad, CA). The 3.1 kb fragment was mapped with selected restriction enzymes. The appropriately sized and overlapping *Xba*I and *Nsi*I subfragments were subcloned into pBluescript II-SK(+) (Stratagene, CA) and sequenced.

### Isolation and sequencing of 5′ end of TNNT3 gene

The 5′ end of TNNT3, including exon one, promoter and 5′ flanking region, was isolated using the standard protocol accompanying the GENOME-WALKER kit (Clonetech). Briefly, two antisense primers were used as the gene specific primers. These primers were designed from the same alignment of a human EST (GenBank Accession No. AA194223) with the mouse and rat fast skeletal TnT cDNA sequences, as described for isolating TNNT3 intron 1 (see above). The primer pDW1 (5′-ctgcgggtcgagtgtgagaag-3′) was used in the primary PCR. The primer pDW2 (5′-agggctggagctccctgtgg-3′) was used in the secondary 'nested' PCR. A two-temperature PCR amplification method was used for both reactions. The primary PCR was performed for seven cycles: 94 °C for 25 s; 72 °C for 4 min; followed by 32 cycles of 94 °C for 25 s, 67 °C for 4 min; followed by 67 °C for an additional 4 min after the final cycle. After amplification, we obtained a single 1.7 kb and a single 350 bp fragment for each of the two human genomic DNA templates (digested with different restriction enzymes) supplied by the vendor. 2 µl aliquots were used for ligation into the TA cloning vector, following the standard protocol for ligation as supplied by the vendor (Invitrogen). Recombinant plasmids were analysed by restriction enzyme digestion and agarose gel electrophoresis. Single plasmids containing the 1.7 kb and 350 bp inserts were isolated using the Qiagen Midi Prep protocol, as supplied by the manufacturer. Both plasmids were sequenced bidirectionally, using m13 forward and m13 reverse universal primers. To augment the sequencing of the 1.7 kb fragment, the plasmid was subjected to exonuclease III digestion using the Exo-Size kit from New England Biolabs. The standard protocol was followed using *Nsi*I as the 3′ overhang protected end, and

**Figure 1.** Multiple alignment of mouse (L49471), rat (M15202) and human (AA194223) fast skeletal TnT cDNA sequences, representing the boundary region of exons 1 and 2. Gaps in alignment are indicated by — . The start codon located in exon 2 is in position 81–83. The first nucleotide in the rat transcript is number 1

Copyright © 2003 John Wiley & Sons, Ltd. 

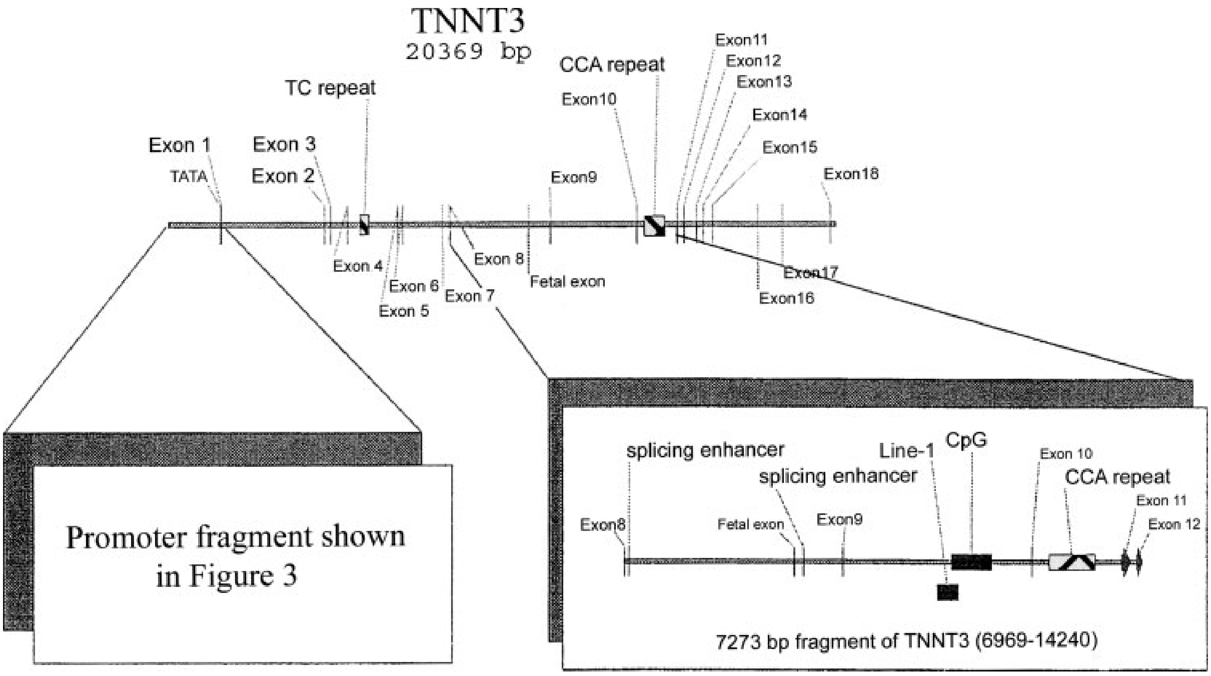

**Figure 2.** Schematic representation of TNNT3. Constitutive exons are marked above the gene, while alternatively spliced exons are indicated below the line representing TNNT3. The positions of splicing enhancers, TC and CCA repeats and a LINE-1 element are also shown. For further details, see text

*Xho*I as the 5′ overhang exonuclease III susceptible end. Six pools of decreasing insert sizes were religated using T4 DNA ligase supplied with the kit. Individual white colonies were selected. The inserts present in the plasmids ranged in size from 1.7 kb (control) to a few hundred base pairs. The inserts from two selected plasmids (~1.4 kb and ~800 bp) were sequenced using m13 forward and m13 reverse universal primers.

### Computer-aided sequence analysis

We used the gapped BLAST program (Altschul *et al.*, 1997) for searching in GenBank at its World Wide Web server (**http://www.ncbi.nlm.nih.gov/**). For sequence analysis, the Genetics Computer Group (GCG; Madison, WI) program package version 8.0 was used. Putative transcription factor binding sites in the promoter region of TNNT3 were identified using the MAP program from the GCG package and a muscle-specific subset of GCG Transcription Factor Database. The putative muscle-specific splicing enhancers (MSEs) in TNNT3 were identified by a FASTA search using the reported MSE sequence in the chicken cTNT gene (Ryan and Cooper, 1996).

### Results

### Nucleotide sequence and genomic organization

The structure and complete sequence of the human fast skeletal TnT gene is reported in this study. The TNNT3 gene is encoded by 19 exons spaced by 18 introns within approximately 19 kb of genomic DNA (Figure 2). We determined the sequence of 20 369 nucleotides of TNNT3 spanning from −1.7 kb relative to the transcriptional start site and including the 3′ untranslated region. We identified all the 19 exons in the TNNT3 gene based on the published cDNA sequence of the human fast skeletal $\beta$ TnT (Wu *et al.*, 1994) and the rat fast skeletal TnT gene (Breitbart and Nadal-Ginard, 1986). The sizes of these exons range from 12 bp to more than 160 bp. The length of each intron and exon, the position and main features of each exon, and the sequences of exon–intron boundaries are shown in Table 1. The complete sequence of TNNT3 (GenBank Accession No. AF026276) is available as supplementary material Insert URL[INSERT URL HERE].

Regarding the numbering of exons, for more convenient comparisons we followed the convention

Copyright © 2003 John Wiley & Sons, Ltd. 

**Table 1.** Exon/intron structure of TNNT3

| Exon | Position | Size (bp) | Acceptor | Exon | Donor | Intron (bp) | Features |
|---|---|---|---|---|---|---|---|
| 1 | 1–52 | 52 | | C...CAG | GTGGGT... | 3090 | 5′ UTR |
| 2 | 3143–3177 | 35 | ...CTGCAG | A...AGT | GTGAGT... | 156 | 5′ UTR, ATG start |
| 3 | 3334–3347 | 14 | ...CCACAG | T...AGG | GTAAGT... | 493 | Constitutive |
| 4 | 3841–3858 | 18 | ...ATGCAG | A...AAG | GTAATT... | 1517 | Alternative splicing |
| 5 | 5376–5393 | 18 | ...TCTCAG | A...AAG | GTAAGT... | 137 | Alternative splicing |
| 6 | 5531–5551 | 21 | ...CCTCAG | C...AAG | GTATGA... | 1190 | Alternative splicing |
| 7 | 6742–6753 | 12 | ...GTGAAG | T...CAG | GTACGT... | 214 | Alternative splicing |
| 8 | 6968–6982 | 15 | ...CTGCAG | A...AAG | GTMCGC... | 2394 | Alternative splicing |
| Foetal | 9377–9400 | 24 | ...TGGAAG | A...AAG | GTAAGG... | 657 | Alternative splicing |
| 9 | 10058–10076 | 19 | ...GACCAG | A...CAA | GTGAGT... | 2624 | Constitutive |
| 10 | 12701–12746 | 46 | ...CTTCAG | A...GAT | GTAAGT... | 1206 | Constitutive |
| 11 | 13953–14069 | 117 | ...CCACAG | G...ATC | GTAAGT... | 93 | Constitutive |
| 12 | 14163–14240 | 78 | ...CTGCAG | G...GCG | GTTAGG... | 321 | Constitutive |
| 13 | 14562–14675 | 114 | ...CCACAG | G...AAG | GTGTGT... | 98 | Constitutive |
| 14 | 14774–14883 | 110 | ...CTGCAG | G...GAG | GTGAGT... | 168 | Constitutive |
| 15 | 15052–15142 | 91 | ...GGGGTG | G...GAC | GTGAGT... | 1284 | Constitutive |
| 16($\alpha$) | 16427–16467 | 41 | ...TTCTAG | A...GTT | GTAAGT... | 702 | Alternative splicing |
| 17($\beta$) | 17170–17210 | 41 | ...TCACAG | A...GCA | GTGAGT... | 1417 | Alternative splicing |
| 18 | 18628–(18787) | >161 | ...TTGCAG | C... | | | Constitutive, stop codon, 3′ UTR |

For details, see also text and Figure 2.
The underlined letters indicate non-conserved residues at exon–intron boundaries according to the acceptor site consensus YYNCAG—G and the donor site consensus AG—GTRAGT (Senapathy *et al.*, 1990).

established for numbering the rat fast skeletal TnT gene (Breitbart and Nadal-Ginard, 1986). Accordingly, the embryonic isoform-specific 'foetal' exon, which was proposed subsequently (Briggs and Schachat, 1993), is not numbered. The putative promoter at the 5′ flanking region, the nucleotide sequence of exon 1 and upstream region to position −262 is shown in the inset (Figure 3). The genomic sequence further upstream to −1.7 kb is reported in the supplementary material. This is the first description of the 5′ flanking region for a gene coding for any TnT isoform and should provide new information about the regulation of the gene. A canonical TATA-box is present at −36 from the cap site. We find a typical CAAT-box at position −67. Notably, there are several potential E-boxes (target sequences for basic helix–loop–helix transcription factors, such as MyoD) and MEF-2 binding sites. It should be noted that only those elements that match perfectly to the binding sites of the above-mentioned transcription factors are shown in Figure 3. It is currently believed that these transcription factors are responsible for muscle-specific activation of several contractile protein-specific genes during skeletal myogenesis (Firulli and Olson, 1997). Therefore, it is suggested that these *cis* elements, together with their *trans* factors, may play a role in the transcriptional regulation of TNNT3.

### Hypermethylated fragment and LINE-1 element in intron 9

A gapped BLAST nucleotide database search (Altschul *et al.*, 1997) using the nucleotide sequence of TNNT3 identified a human genomic DNA sequence, clone 5a2 (GenBank Accession Nos Z61 863 and Z61 862), which corresponds to the 13 151–13 714 region of intron 9 of the TNNT3 gene. Clone 5a2 was not identified as part of TNNT3 by the depositors of the sequence. This DNA fragment was cloned by affinity purification of putative CpG island genomic DNA using the methyl-cytosine binding protein MeCp2 (Cross *et al.*, 1994). We carefully examined the frequency of CG dinucleotides in the TNNT3 gene using the STATPLOT and WINDOW programs of the GCG program package. We found that neither the 13 151–13 714 region nor any other part of the TNNT3 gene contained a sufficient amount of CG dinucleotides to be classified as a CpG island. Indeed, CG dinucleotides are under-represented in the TNNT3 gene sequence. This reflects the characteristics of non-CpG island sequences in the human genome (Cross and Bird, 1995).

Copyright © 2003 John Wiley & Sons, Ltd. 

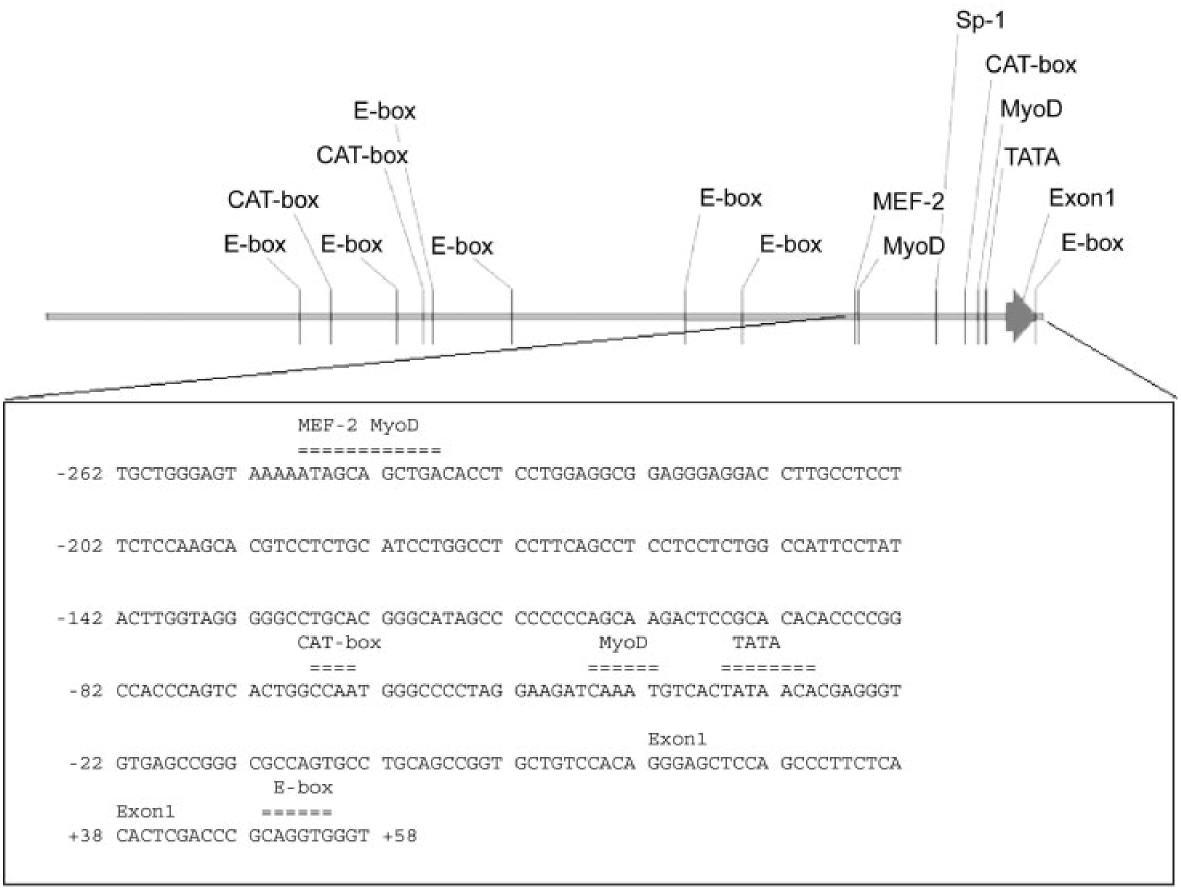

**Figure 3.** Promoter region of TNNT3. Putative *cis* regulatory sequences, the TATA box, and exon 1 are indicated. Only those elements that match perfectly to binding sites of transcription factors acting in muscle are shown. For further details, see text

However, further examination of the intron 9 sequence revealed the presence of a cryptic long interspersed nuclear element (LINE-1) overlapping the clone 5a2 region (Figure 2). A similar cryptic LINE-1 sequence is also present in the rat fast skeletal TnT gene at a homologous position. It is believed that suppression of parasitic sequence elements is the primary function of cytosine methylation (Yoder *et al.*, 1997). This may explain the presence of hypermethylation in intron 9, because it is currently believed that LINE-1 elements are degenerate copies of transposable elements (Boeke, 1997; Sassaman *et al.*, 1997; Smit, 1996).

### Comparison of TNNT3 with mammalian homologues

Comparison of the rat orthologue with TNNT3 by dot-plot analysis reveals a high degree of similarity and co-linearity between the two genes (Figure 4). However, there are two short regions in the human gene that are not co-linear. Self-comparison of TNNT3 using dot-plot analysis (Figure 5) reveals that the interruptions in co-linearity are due to the presence of simple sequence repeats in the human TNNT3 gene. One repeat (TC repeat, Figures 2, 4 and 5), is located between nucleotides 4228 and 4498 relative to the transcription initiation site, while the other repeat (CCA repeat) lies between nucleotides 12 938 and 14 028.

TNNT3 possesses a weaker but considerable similarity to the rat cardiac TnT gene (Jin *et al.*, 1992). There appears to be no homologue of exon 12 of the rat cardiac TnT gene in comparison with TNNT3. Exons 6 and 7 of TNNT3 are similar to a single exon (exon 5) in the rat cardiac TnT gene. Also, there is no homologue of exon 17 of

Copyright © 2003 John Wiley & Sons, Ltd.

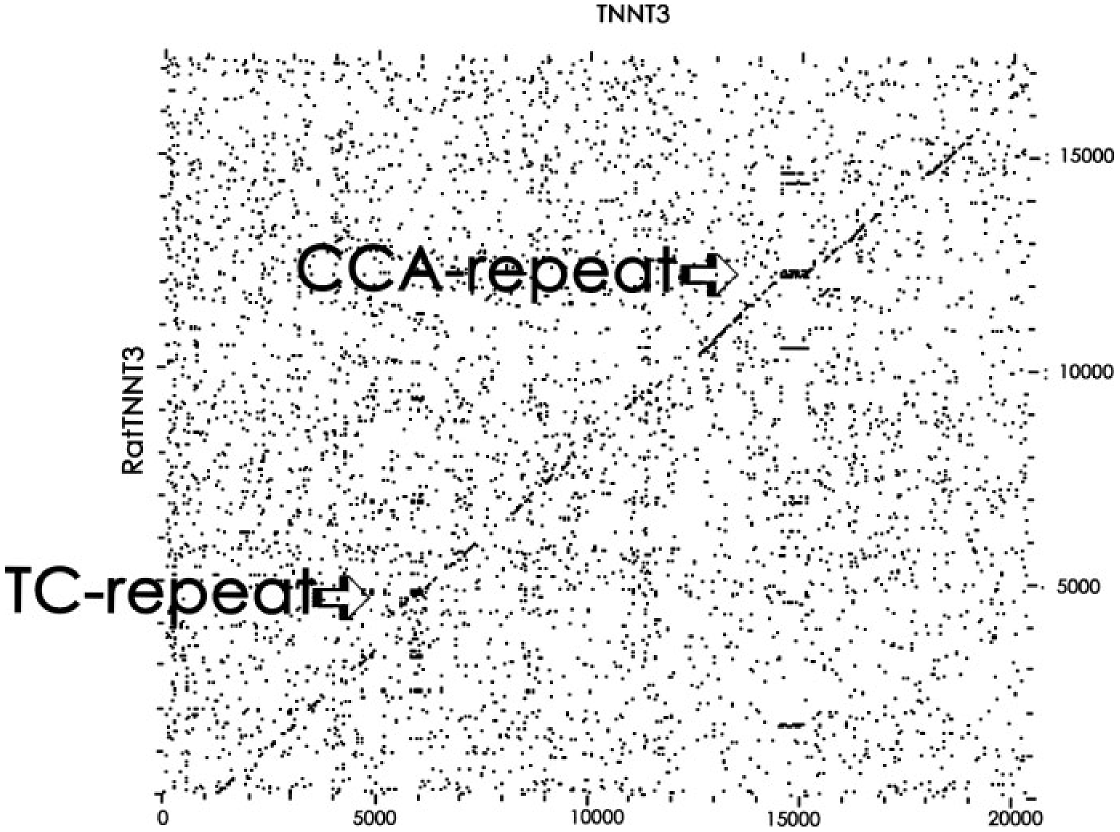

**Figure 4.** Dot-plot analysis of the similarity between TNNT3 and its rat homologue. Coordinates of common points between the two sequences obtained by matrix analysis are plotted. CCA and TC repeats are indicated

TNNT3 in the cardiac TnT gene. The cardiac TnT paralogue possesses only a constitutive exon (exon 15) in place of the alternatively spliced exons 16 and 17 of TNNT3.

Several lines of evidence suggest that the alternatively spliced $\alpha$ (16) and $\beta$ (17) exons of TNNT3 are the result of exon duplication: (a) they are of equal length; (b) the nucleotide sequence shows high similarity (47.5% identity; Figure 6A); and (c) the amino acid sequences encoded by them show 60% identity (Figure 6B). Since the genetic code is degenerate, this nucleotide sequence homology (Figure 6A) strongly suggests that the $\alpha$ and $\beta$ exons originated by exon duplication.

### Exon and intron phasing in TNNT3: implications on the evolution of the gene

The phase classes of introns according to their position relative to the reading frame of the genes containing the introns have been defined (Sharp, 1981) as described below: (a) introns present in the 5′ or 3′ untranslated regions of transcripts; (b) introns lying between the first and second nucleotides of a codon (phase 1 intron); (c) introns lying between the second and third nucleotides of a codon (phase 2 intron); (d) introns lying between two codons (phase 0 intron). Introns are considered homologous if they can be shown to be derived from the same ancestral intron. However, homology of some introns may be recognized simply from their position in the sequence (Patthy, 1987). For example, they lie in the same position of the aligned sequences of homologous genes or gene segments and split the reading frame in the same phase. Intron 16 of TNNT3 splits the reading frame in the same phase as its rat, avian, insect and nematode homologues. Furthermore, as shown in Figure 7, equivalents of intron 16 lie in a homologous position in the amino acid sequence alignments of TnT isoforms. All of the conserved intron 16 homologues are located in the region encoding for the

Copyright © 2003 John Wiley & Sons, Ltd. 

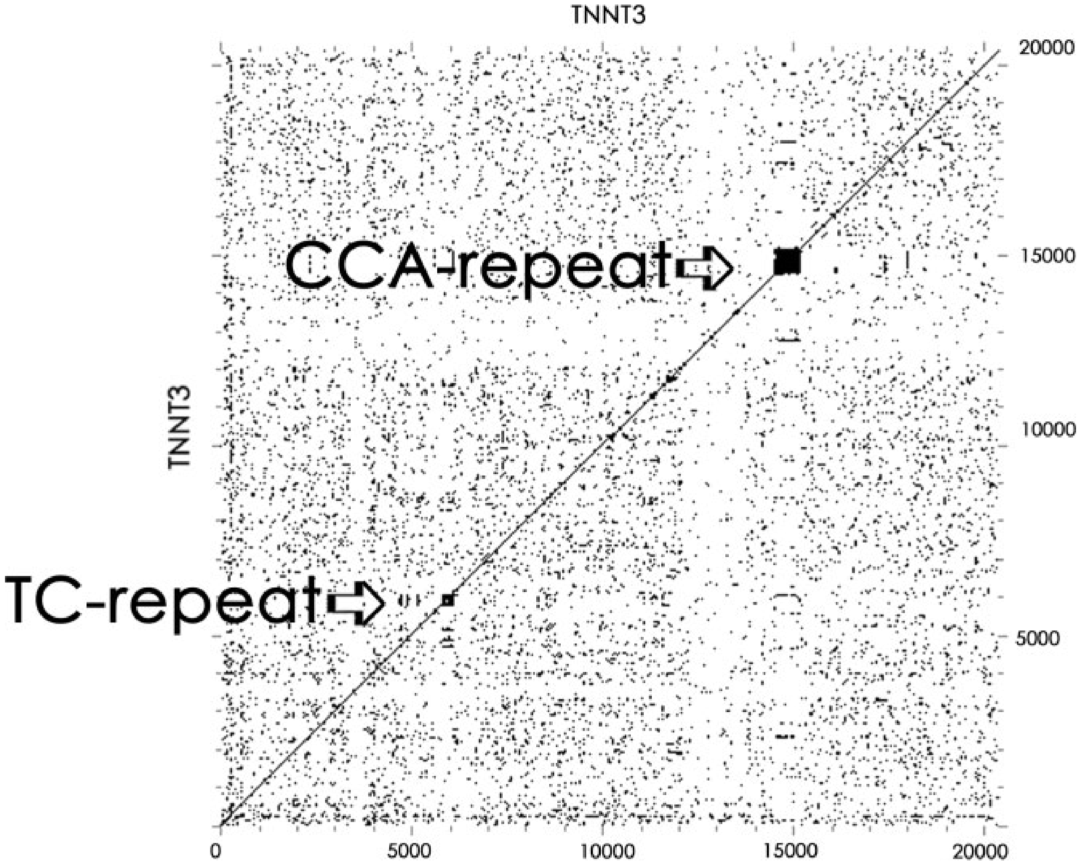

**Figure 5.** Dot-plot analysis of the self similarities in TNNT3. Coordinates of common points obtained by matrix analysis are plotted. CCA and TC repeats are indicated

HR domain of TnT (cf. Figure 7 with amino acid sequences of the conserved HR domains of TnT isoproteins shown in Stefancsik *et al.*, 1998). The HR domain represents the most conserved region in the entire poypeptide sequences of TnT isoproteins present in phylogenetically distant species (Stefancsik *et al.*, 1998). The position of homologous introns may differ because of splice-junction sliding. Thus, the correct reading frame is only preserved if the original intron phase is maintained. Also, evolutionary selection generally prefers the conservation of intron phase classes.

Exon shuffling is believed to be very important for the molecular evolution and diversification of proteins, by allowing recombination of protein modules (Gilbert, 1978; Long *et al.*, 1995). Exons, or exon sets, can be classified with respect to the position of their 5′ and 3′ splice junctions in the reading frame. Exons with symmetrically phased flanking introns differ markedly in their versatility in exon shuffling. In particular, only those exons that have introns of the same phase class at the 5′ and 3′ ends (symmetrical exons of classes 1–1, 2–2, 0–0) can be inserted into introns of the same phase class. They can undergo tandem duplication into adjacent introns, or can be deleted by intronic recombination without disrupting the reading frame (Patthy, 1987). Analysis of the phase distribution of introns in TNNT3 and other available TnT genomic sequences (Tables 2 and 3) shows the following features: as is apparent from the distribution of intron phases, there is a high degree of similarity among vertebrate cardiac and skeletal TnT genes (Table 2). However, we also find some differences, e.g. the number of phase ‘0’ introns, located in exons encoding for short acidic peptides at the amino-terminus of TnT, differ between TnT isoform genes. These exons are also flanked by symmetrically phased introns (Figure 8). To identify symmetrically phased exons, we also plotted

Copyright © 2003 John Wiley & Sons, Ltd.

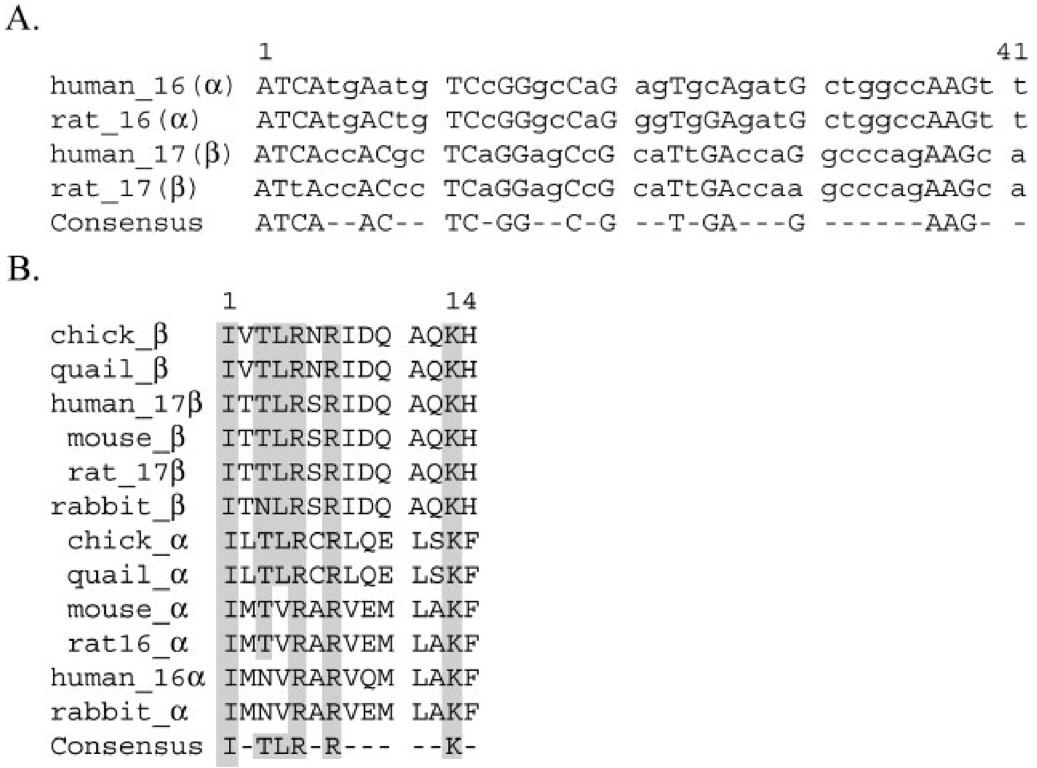

**Figure 6.** Comparison of the alternatively spliced $\alpha$ and $\beta$ exons of vertebrate species. (A) Nucleotide sequence alignment of exons 16 and 17 of the rat and human fast skeletal troponin T genes. Consensus positions in the alignment are marked by capitalization. (B) Alignment of the peptides encoded by the $\alpha$ and $\beta$ exons. Consensus positions are shaded the phase changes in TNNT3. We defined the phase change ($\Delta\Phi$) for introns as the difference between phase values of introns following and preceding a given exon: $\Delta\Phi = \Phi_{n+1} - \Phi_n$, where $n$ is the serial number of an intron following the $n$th exon; $n \in \{1, 2, \ldots\ N - 1\}$; $N$ = number of introns. Exons 4–8 and the 'foetal' exon that precedes exon 9, exhibit 0 phase change. Interestingly, all of these exons are alternatively spliced. Exons 11–13, which are constitutively spliced, also show 0 phase change. Thus, both of these exon groups (4–8 and foetal; 11–13) represent potential products of exon shuffling. Exon 17 (the embryo or foetal specific $\beta$ exon) is also symmetrically phased and shows alternative splicing. By comparing the intron phase distribution of vertebrate and invertebrate TnT genes, it appears quite unlikely that the HR domain in TnT genes originated by exon shuffling (Tables 2 and 3), because of the presence of non-symmetrically phased exons. In contrast, similar analysis indicates that TnI genes almost exclusively consist of symmetrically phased exons flanked by phase 0 introns (unpublished observation), suggesting that exon shuffling also played an important role in their evolution (Patthy, 1987).

### Potential regulatory elements for alternative splicing

An exonic splicing enhancer that is required for the inclusion of the alternatively spliced exon 5 of the chicken cardiac TnT (cTnT) gene in mRNA has been previously reported (Ramchatesingh *et al.*, 1995). Interestingly, a sequence element similar to the above-mentioned nine nucleotide splicing enhancer motif (GAGGAGAA) is also present in the 'foetal' exon of TNNT3 (supplementary material). The alternative exon 5 of the chicken cardiac TnT gene is included in mRNA from embryonic skeletal and cardiac muscle and excluded in mRNA from the adult tissues. A similar embryonic mRNA-specific inclusion is observed for the 'foetal' exon of TNNT3 (Briggs *et al.*, 1994). Another feature related to the potential regulation

Copyright © 2003 John Wiley & Sons, Ltd. 

```

TNNT3 (human fast skeletal TnT)

**Figure 7.** Conserved intron position (||) in TnT genes. Sources of DNA sequences: *TNNT3*, present work; *RATTNT*, Breitbart and Nadal-Ginard (1986); *RATCTTG*, Jin *et al.* (1992); *CHKTNTC*, Cooper and Ordahl (1985); *DMTROPT*, Fyrberg *et al.* (1990); *CeTNT1*, Myers *et al.* (1996); *CeTNT2*, GenBank Accession No. 746561; *CeTNT3*, GenBank Accession No. 861386; *CeTNT4*, GenBank Accession No. 2736369

of alternative splicing of chicken cardiac TnT is that the introns flanking exon 5 of the chicken cTnT gene contain muscle-specific splicing enhancers (MSEs) acting in a positive manner to mediate the embryonic splicing pattern of exon inclusion (Ryan and Cooper, 1996). We have identified a homologue of this intronic MSE motif in the intron upstream (following exon 8), and downstream (preceding exon 9) from the 'foetal' exon of TNNT3 (Figures 2 and 9). This homologue of the MSE motif in TNNT3 shows 77.8% identity with the reported chicken cardiac MSE sequence. Similar MSE motifs are also found in homologous position in the rat cardiac and fast skeletal TnT genes (Figure 9). In view of these observations, it is suggested that the homologue of the MSE element in TNNT3 may play a role in regulating the inclusion of the 'foetal' exon into human foetal and embryonic TnT mRNAs.

### Discussion

In this report we describe the structure and complete nucleotide sequence of the TNNT3 gene. This

Copyright © 2003 John Wiley & Sons, Ltd.

**Table 2.** Intron phasing of vertebrate TnT genes

| Intron No./phase | | 1 | 2 | 3 | 4 | 5 | 6 | 7 | 8 | 9 | 10 | 11 | 12 | 13 | 14 | 15 | 16 | 17 | 18 |
|---|---|---|---|---|---|---|---|---|---|---|---|---|---|---|---|---|---|---|---|
| Human TNNT3 | 2 | | X | | | | | | | | X | | | | | X | | X | X |
| | 1 | | | X | X | X | X | X | X | X | | | | | | | | | |
| | 0 | n | | | | | | | | | | X | X | X | X | | **XI** | | |
| Rat fast skeletal TnT | 2 | | X | | | | | | | | X | | | | | X | | X | X |
| | 1 | | | X | X | X | X | X | X | X | | | | | | | | | |
| | 0 | n | | | | | | | | | | X | X | X | X | | **XI** | | |
| Rat cardiac TnT | 2 | | X | | | | | X | | | | | | X | | X | | | |
| | 1 | | | X | X | X | X | | | | | X | X | | | | | | |
| | 0 | n | | | | | | | X | X | X | | | | **XI** | | | | |
| Chicken cardiac TnT | 2 | | X | | | | | | | X | | | | | X | | X | X | |
| | 1 | | | X | X | X | X | X | X | | | | | | | | | | |
| | 0 | n | | | | | | | | | X | X | X | X | | **XI** | | | |

For details, see also text. n, intron in non-coding region; XI, intron located in the region coding for HR domain. Sources of DNA sequences: human TNNT3, present work; rat fast skeletal TnT, Breitbart and Nadal-Ginard (1986); rat cardiac TnT, Jin *et al.* (1992); chicken cardiac TnT, Cooper and Ordahl (1985).

**Table 3.** Intron phasing of invertebrate TnT genes

| Intron No./phase | | 1 | 2 | 3 | 4 | 5 | 6 | 7 | 8 | 9 | 10 | 11 | 12 | 13 | 14 | 15 | |
|---|---|---|---|---|---|---|---|---|---|---|---|---|---|---|---|---|---|
| *Drosophila* TnT | 2 | | X | X | X | | | | | | | | | | | | |
| | 1 | | | | | | | | | | X | | | | | | |
| | 0 | | | | | X | X | XI | X | X | | | | | | | |
| *C. elegans* TnT-1 | 2 | | X | X | | | | | | | | | | | | | |
| | 1 | | | | | | | | | | | | | | | | |
| | 0 | | | | X | X | XI | | | | | | | | | | |
| *C. elegans* TnT-2 | 2 | | | X | | | X | | | | | | | | | | |
| | 1 | | X | | | | | | | | | | | | | | |
| | 0 | | | | X | XI | | X | | | | | | | | | |
| *C. elegans* TnT-3 | 2 | | | X | X | X | X | | | X | | | | | | | |
| | 1 | | | | | | | | | | | | | | | X | X |
| | 0 | | X | | | | | X | X | | X | XI | X | X | X | | |
| *C. elegans* TnT-4 | 2 | | X | X | | | | | | | | | | | | | |
| | 1 | | | | | | | | | | | | | | | | |
| | 0 | | | | XI | X | X | | | | | | | | | | |

For details, see also text. XI, intron located in the region coding for the HR domain. Sources of DNA sequences: *Drosophila* TnT, Benoist *et al.* (1998), Fyrberg *et al.* (1990); *C. elegans* TnT-1, Myers *et al.* (1996); *C. elegans* TnT-2, GenBank Accession No. 746561; *C. elegans* TnT-3, GenBank Accession No. 861386; *C. elegans* TnT-4, GenBank Accession No. 2736369.

is the first report on the characterization of any human striated TnT isoform gene. Furthermore, we have attempted to study the contribution of genomic correlates of TNNT3 to several interesting features of TnT biology that have relevance to the structure and function of the polypeptide and the evolution of the gene.

The structure of the promoter and sequence of the 5′ regulatory regions of the TNNT3 gene are the first description of the 5′ flanking region for any TnT gene. This information will be useful in understanding how the tissue-specific expression of TnT isoform genes in muscle cells is regulated. The presence of MyoD and MEF-2 binding sites at the TNNT3 promoter suggests a regulatory role, because these transcription factors are essential for muscle-specific activation of myofibrillar protein genes (Firulli and Olson, 1997). However, further experimental evidence will be required to elucidate the role for any of the identified potential *cis*

Copyright © 2003 John Wiley & Sons, Ltd. 

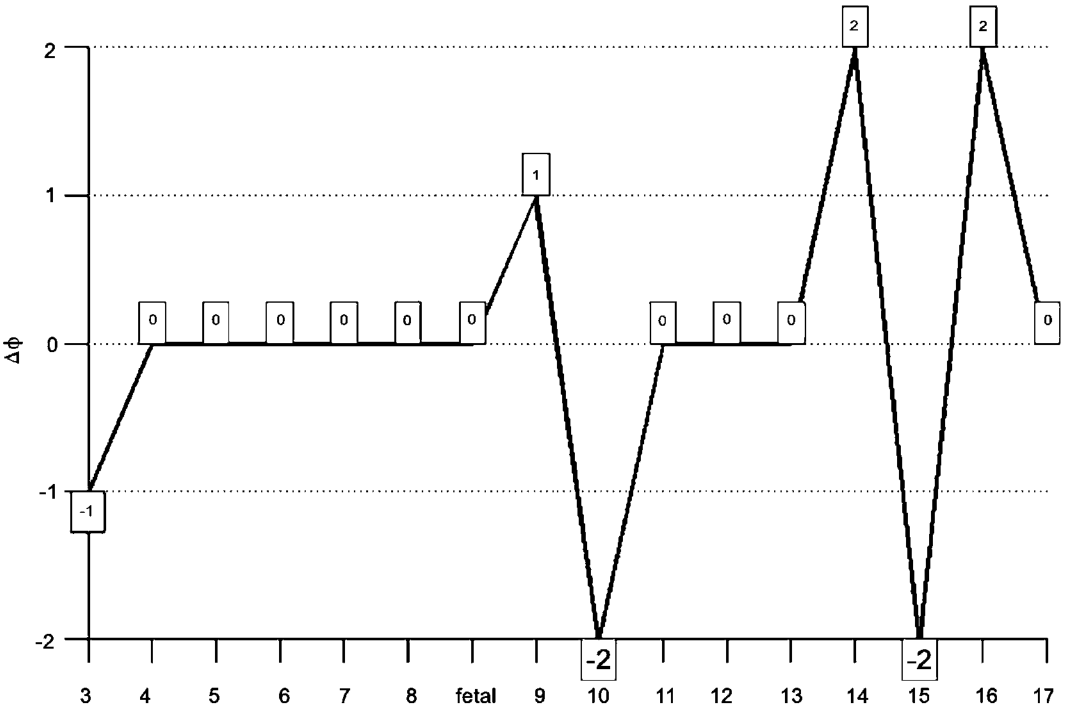

**Figure 8.** Phase change of flanking introns in TNNT3. Phase change for exons 1, 2 and 18 are not applicable, because of the presence of non-coding regions (5′ and 3′ UTRs). The HR domain is encoded by exons 14–17. Phase change ($\Delta\Phi$) for introns is defined as the difference between phase values of introns following and preceding a given exon: $\Delta\Phi = \Phi_{n+1} - \Phi_n$, where $n$ is the serial number of an intron following the $n$th exon; $n \in \{1, 2, \ldots N - 1\}$; $N$ = number of introns. Note that symmetrically phased exons possess 0 phase change. For explanation of intron/exon phasing, see also text

```
1  ggg-----tcggtgtgtcctgtg------------cctttccctgct-----tgggaaacgc
2  --g--t--tgtttctgtccc-tg-------------ctttcc-tgctcctcctgggctctg-
3  tt---tactctatctgtcca-tgtctgtctgtcttcctttc----tc--cctgg-------
4  cctcctcctccctctgtcct-----------------ctttctct--tc--cctggcacgg--
```

**Figure 9.** Homology of the muscle-specific splicing enhancer (MSE) elements in vertebrate striated muscle TnT genes. 1, Chicken cardiac TnT; 2, rat cardiac TnT; 3, rat fast skeletal TnT; 4, human TNNT3. Nucleotides showing sequence similarity are shaded. For details see also text regulatory elements. The sequence information provided in this report is a prerequisite for carrying out studies on the biological role of these presumptive regulatory elements. In addition, a detailed understanding of the genomic correlates that are involved in regulating the tissue-specific expression of striated muscle TnT isoforms, has clinical relevance to human muscle disorders of genetic origin. Mutations in cardiac troponin T account for approximately 15% of human familial hypertrophic cardiomyopathy cases (Watkins *et al.*, 1995). Information on the 5′ flanking region of the TNNT3 gene may lead to the development of therapeutic strategies based on the ectopic activation of the fast skeletal TnT gene in the cardiac muscle of individuals affected by familial hypertrophic cardiomyopathy.

Comparison of the human and rat fast skeletal TnT genomic sequences reveals high nucleotide sequence similarity, with the exception of two repeat regions comprised of 'CCA' and 'TC' repeats. We identified a LINE-1 element in TNNT3. A homologous sequence is also present in the rat fast skeletal TnT gene, indicating that the insertion of this sequence element occurred in a common ancestral line of primates and rodents. The

Copyright © 2003 John Wiley & Sons, Ltd. 

presence of a cryptic LINE-1 element in intron 9 may explain the hypermethylation in the neighbouring genomic region, since defective transposable elements (Smit, 1996) are believed to be the targets of DNA methylation and silencing (Walsh and Bestor, 1999; Yoder *et al.*, 1997). The 5a2 region in intron 9, which was cloned by Cross *et al.* (1994), was believed to be part of a CpG island. However, our sequence analysis did not identify any CpG island in TNNT3. A possible explanation for the high affinity of the 5a2 region for the MeCp2 column used for cloning this fragment could be hypermethylation or unusual DNA structure (Cost and Boeke, 1998), presumably due to the presence of a LINE-1 element adjacent to it.

The identification of MSEs acting as *cis* regulatory elements in tissue and developmentally regulated manner in the cardiac TnT gene was reported previously (Ryan and Cooper, 1996). We also find homologous MSE motifs in the introns upstream and downstream of the human 'foetal' exon. Moreover, the constitutive splicing enhancer required for the inclusion of the alternative exon 5 of the chicken cTnT gene (Ramchatesingh *et al.*, 1995) has a homologue in the 'foetal' exon of TNNT3. These observations suggest that the regulatory pathway for the developmental pattern of alternative splicing in troponin T genes has been conserved from birds to humans. However, this view needs to be supported by experimental evidence that the homologous MSE elements of TNNT3 play a regulatory role in the alternative splicing of the 'foetal' exon.

The genomic correlates of TNNT3 reported in this study provide some insight into the structure, function and pattern of sequence conservation of TnT and its two proteolytic fragments, the C-terminal T2 (amino acid residues ∼160–260) and the N-terminal T1 (amino acid residues ∼1–159). The T2 segment together with TnC and TnI constitutes the 'globular head' region of Tn complex. It is involved in intersubunit interactions with TnC and TnI and plays a role in the $Ca^{2+}$ regulation of vertebrate striated muscle contraction, particularly in the 'information transfer' process, presumably in the order TnC → TnI → TnT → Tm-actin (Perry, 1998; Gordon *et al.*, 2000; Jha and Sarkar, 1998). The high degree of sequence conservation in T2 segments among the vertebrate inter-and intraspecies isoforms reflects three features, the first being that the amino acid sequences in T2 segments are primarily derived from constitutively spliced exons located in the 3′ end of the gene (Table 1; Breitbart and Nadal-Ginard, 1986). The second feature is that, in contrast to the multiple alternatively spliced 5′ exons, there is a single alternative splice site in the 3′ segment of TNNT3 (Table 1). This involves exons 16 ($\alpha$) and 17 ($\beta$), which code for a similar but non-identical 14 amino acid peptides (Figure 6). These two exons are spliced in a developmentally regulated and mutually exclusive manner (Perry, 1998; Wu *et al.*, 1994) in both birds and mammals. The third feature is the evolutionarily highly conserved HR domains (residues ∼179–241 in skeletal fast TnT) previously identified by us in phylogenetically distant TnT sequences are also located in the T2 segment (Stefancsik *et al.*, 1998). Interestingly, the $\alpha$ and $\beta$ exons are located at the distal C-terminal part of the conserved HR domain. This suggests that binary TnI–TnT interactions mediated by HR domains may be developmentally regulated in embryonic or foetal stages in both birds and mammals. The sequence conservation in the T2 segments of vertebrate TnT isoforms reflects the stringent constraints for maintaining the tertiary structure of the C-terminal half of the polypeptide that is required for intersubunit interactions and $Ca^{2+}$-dependent binding of Tm. This view is also strongly supported by the genomic correlates of TNNT3 (Figure 2, Table 1).

The T1 segment of vertebrate TnT isoforms anchors the Tn complex to Tm-actin. It is also responsible for the cooperativity of the $Ca^{2+}$ activation of the acto-myosin ATPase (Perry, 1998; Gordon *et al.*, 2000; Schaertl *et al.*, 1995). The T1 segments contain a hypervariable N-terminal region (residues 1–41 in skeletal fast; 1–45 in cardiac and slow skeletal TnT) that flanks the central conserved region of about 160 amino acids (residues 42–204 in fast and 46–204 in slow and cardiac TnT) present in mammalian TnT isoforms (Wu *et al.*, 1994). This conserved region constitutes the Tm binding site. It is partly located in the T1 fragment (residues ∼42–159), whereas the TnI binding site involving the conserved HR domain is restricted to the T2 segments. The N-terminal hypervariable region of T1 segments spans the head-to-tail overlap region of contiguous Tm molecules. This region may play a modulatory 'fine tuning' role in the interaction with Tm, leading to variation in $Ca^{2+}$ sensitivity of individual muscle fibres (Briggs and

Copyright © 2003 John Wiley & Sons, Ltd. 

Schachat, 1989; Tobacman and Lee, 1987; Malnic *et al.*, 1998). The alternative splicing involving the 5′ mini exons (exons 4–9) in a combinatorial manner generates the variable N-terminal sequence divergence observed in mammalian skeletal TnT isoforms (Breitbart and Nadal-Ginard, 1986; Wu *et al.*, 1994). Furthermore, together with the single alternative splice site involving exons 16 and 17 at the 3′ end of the gene, the 'foetal' exon located between exons 8 and 9, contributes to the pattern of an additional subset of developmentally regulated isoforms of vertebrate skeletal fast TnT.

The exon phasing of TNNT3 suggests that the alternatively spliced 5′ exons (exons 4–foetal), as well as exons 11–13 and 17, may have evolved as a result of exon shuffling (Figure 8). The introns flanking the exons encoding for the HR domain of TnT do not possess symmetrically phased exons. Thus, this region may not have evolved by exon shuffling. Indeed, the HR domain in TnT isoforms represents the most ancient part of the TnT polypeptide. This view is supported by the presence of a highly conserved intron position that is located in the HR encoding genomic region and also lies in a homologous position in the amino acid alignment from TnT sequences from both protostomes and deuterostomes (Stefancsik, 1999).

The evolutionarily conserved HR domains present in phylogenetically distant TnT and TnI sequences show similarity at a statistically significant level. We suggested that these domains may have a common ancestry (Stefancsik *et al.*, 1998). Examination of exon phasing suggests that the presumptive 'ancestral genes' of TnT (see also Results) and TnI (Patthy, 1987; our unpublished results) have diversified by exon shuffling. For TnT, this process may have involved the generation of additional domains necessary for interaction with other thin filament proteins such as actin, Tm and TnC, as well as the modulatory hypervariable N-terminal region. For TnI, this process may have generated the multiple sites involved in interaction with actin and TnC. For TnT alternative splicing of exons at both the 5′ and 3′ regions of the gene, together with the developmentally regulated expression of 'foetal' and $\alpha/\beta$ exons, provides an additional complex mechanism for generating distinct sets of isoproteins. TnI, on the other hand, shows only tissue-specific isoforms, e.g. fast and slow skeletal and cardiac, and these are not regulated by alternative splicing. It appears that alternative splicing of the TnT gene developed before the divergence of mammalian and avian systems, since this phenomenon is observed in both birds (Hastings *et al.*, 1985; Butcher *et al.*, 1989) and mammals (Medford *et al.*, 1984). These features of TnT and TnI, at both the genomic and polypeptide levels, reflect their distinct role in the 'information transfer' process during $Ca^{2+}$ regulation of vertebrate striated muscle contraction.

### Note

The nucleotide sequence of TNNT3 can be found at **http://www3.interscience.wiley.com/cgi-bin/jabout/77002016/suppmat/index.html**

### Acknowledgments

We thank Drs P. K. Jha, S. Sachdev, P. C. Leavis, T. Chaudhuri, M. Mukerjea and S. Mukhopadhyay for many helpful discussions. We also thank Ms Jacqueline Penn and Mr Paul Morrison for help in the preparation of the manuscript and figures. This work was supported by grants from the National Institutes of Health (P01-HD23681), the American Heart Association and funds from the Tufts University School of Veterinary Medicine. This publication is dedicated to the memory of Professor Amar Nath Bhaduri.

### References

Altschul SF, Madden TL, Schaffer AA, *et al.* 1997. Gapped BLAST and PSI-BLAST: a new generation of protein database search programs. *Nucleic Acids Res* **25**: 3389–3402.

Barlow DP. 1995. Gametic imprinting in mammals. *Science* **270**: 1610–1613.

Benoist P, Mas JA, Marco R, Cervera M. 1998. Differential muscle-type expression of the *Drosophila* troponin T gene. A 3-base pair microexon is involved in visceral and adult hypodermic muscle specification. *J Biol Chem* **273**: 7538–7546.

Boeke JD. 1997. LINEs and Alus — the polyA connection. *Nature Genet* **16**: 6–7.

Breitbart RE, Nadal-Ginard B. 1986. Complete nucleotide sequence of the fast skeletal troponin T gene. Alternatively spliced exons exhibit unusual interspecies divergence. *J Mol Biol* **188**: 313–324.

Breitbart RE, Nguyen HT, Medford RM, *et al.* 1985. Intricate combinatorial patterns of exon splicing generate multiple regulated troponin T isoforms from a single gene. *Cell* **41**: 67–82.

Briggs MM, Maready M, Schmidt JM, Schachat F. 1994. Identification of a foetal exon in the human fast troponin T gene. *FEBS Lett* **350**: 37–40.

Briggs MM, Schachat F. 1993. Origin of foetal troponin T: developmentally regulated splicing of a new exon in the fast troponin T gene. *Dev Biol* **158**: 503–509.

Copyright © 2003 John Wiley & Sons, Ltd.

Bucher EA, De La Brousse FC, Emerson CP Jr. 1989. Development and muscle-specific regulation of avian fast skeletal troponin T isoform expression by mRNA splicing. *J Biol Chem* **264**: 12 484–12 491.

Cooper TA, Ordahl CP. 1985. A single cardiac troponin T gene generates embryonic and adult isoforms via developmentally regulated alternate splicing. *J Biol Chem* **260**: 11 140–11 148.

Cost GJ, Boeke JD. 1998. Targeting of human retrotransposon integration is directed by the specificity of the L1 endonuclease for regions of unusual DNA structure. *Biochemistry* **37**: 18 081–18 093.

Cross SH, Bird AP. 1995. CpG islands and genes. *Curr Opin Genet Dev* **5**: 309–314.

Cross SH, Charlton JA, Nan X, Bird AP. 1994. Purification of CpG islands using a methylated DNA binding column. *Nature Genet* **6**: 236–244.

Dao D, Frank D, Qian N, *et al.* 1998. IMPT1, an imprinted gene similar to polyspecific transporter and multi-drug resistance genes. *Hum Mol Genet* **7**: 597–608.

Farah CS, Reinach FC. 1995. The troponin complex and regulation of muscle contraction. *FASEB J* **9**: 755–767.

Firulli AB, Olson EN. 1997. Modular regulation of muscle gene transcription: a mechanism for muscle cell diversity. *Trends Genet* **13**: 364–369.

Fyrberg E, Fyrberg CC, Beall C, Saville DL. 1990. *Drosophila melanogaster* troponin-T mutations engender three distinct syndromes of myofibrillar abnormalities. *J Mol Biol* **216**: 657–675.

Gilbert W. 1978. Why genes in pieces? *Nature* **271**: 501.

Gordon AM, Homsher E, Regnier M. 2000. Regulation of contraction in striated muscle. *Physiol Rev* **80**: 853–924.

Hastings KEM, Bucher EA, Emerson CP Jr. 1985. Generation of troponin T isoforms by alternative RNA splicing in an avian skeletal muscle: Conserved and divergent features in birds and mammals. *J Biol Chem* **260**: 13 699–13 703.

Henry I, Heyningen VV, Puech A, *et al.* 1993. Reassessment of breakpoints in chromosome 11p15. *Cytogenet Cell Genet* **62**: 52–53.

Jha PK, Sarkar S. 1998. A recombinant monocysteine mutant (Ser to Cys-155) of fast skeletal troponin T: identification by cross-linking of a domain involved in a physiologically relevant interaction with troponins C and I. *Biochemistry* **37**: 12 253–12 260.

Jin JP, Huang QQ, Yeh HI, Lin JJ. 1992. Complete nucleotide sequence and structural organization of rat cardiac troponin T gene. A single gene generates embryonic and adult isoforms via developmentally regulated alternative splicing. *J Mol Biol* **227**: 1269–1276.

Long M, de Souza SJ, Gilbert W. 1995. Evolution of the intron–exon structure of eukaryotic genes. *Curr Opin Genet Dev* **5**: 774–778.

Malnic B, Farah CS, Reinach FC. 1998. Regulatory properties of the $NH_2$- and COOH-terminal domains of troponin T. ATPase activation and binding to troponin I and troponin C. *J Biol Chem* **273**: 10 594–10 601.

Mao C, Baumgartner AP, Jha PK, Huang TH, Sarkar S. 1996. Assignment of the human fast skeletal troponin T gene (TNNT3) to chromosome 11p15.5: evidence for the presence of 11pter in a monochromosome 9 somatic cell hybrid in NIGMS mapping panel 2. *Genomics* **31**: 385–388.

Matsuoka S, Thompson JS, Edwards MC, *et al.* 1996. Imprinting of the gene encoding a human cyclin-dependent kinase inhibitor, p57KIP2, on chromosome 11p15. *Proc Natl Acad Sci USA* **93**: 3026–3030.

McArdle K, Allen TS, Bucher EA. 1998. $Ca^{2+}$-dependent muscle dysfunction caused by mutation of the *Caenorhabditis elegans* troponin T-1 gene. *J Cell Biol* **143**: 1201–1213.

Medford RM, Nguyen HT, Destree AT, Summers E, Nadal-Ginard B. 1984. A novel mechanism of alternative RNA splicing for the developmentally regulated generation of troponin T isoforms from a single gene. *Cell* **38**: 409–421.

Mesnard L, Logeart D, Taviaux S, *et al.* 1995. Human cardiac troponin T: cloning and expression of new isoforms in the normal and failing heart. *Circ Res* **76**: 687–692.

Miyamoto T, Jinno Y, Sasaki T, *et al.* 1996. Genomic cloning and localization to chromosome 11p15.5 of the human achaete-scute homologue 2 (ASCL2). *Cytogenet Cell Genet* **73**: 312–314.

Morgan MJ, Earnshaw JC, Dhoot GK. 1993. Novel developmentally regulated exon identified in the rat fast skeletal muscle troponin T gene. *J Cell Sci* **106**: 903–908.

Myers CD, Goh PY, Allen TS, Bucher EA, Bogaert T. 1996. Developmental genetic analysis of troponin T mutations in striated and nonstriated muscle cells of *Caenorhabditis elegans*. *J Cell Biol* **132**: 1061–1077.

Patthy L. 1987. Intron-dependent evolution: preferred types of exons and introns. *FEBS Lett* **214**: 1–7.

Perry SV. 1998. Troponin T: genetics, properties and function. *J Muscle Res Cell Motil* **19**: 575–602.

Pettenati MJ, Haines JL, Higgins RR, *et al.* 1986. Wiedemann–Beckwith syndrome: presentation of clinical and cytogenetic data on 22 new cases and review of the literature. *Hum Genet* **74**: 123–154.

Qian N, Frank D, O'Keefe D, *et al.* 1997. The IPL gene on chromosome 11p15.5 is imprinted in humans and mice and is similar to TDAG51, implicated in Fas expression and apoptosis. *Hum Mol Genet* **6**: 2021–2029.

Ramchatesingh J, Zahler AM, Neugebauer KM, Roth MB, Cooper TA. 1995. A subset of SR proteins activates splicing of the cardiac troponin T alternative exon by direct interactions with an exonic enhancer. *Mol Cell Biol* **15**: 4898–4907.

Ryan KJ, Cooper TA. 1996. Muscle-specific splicing enhancers regulate inclusion of the cardiac troponin T alternative exon in embryonic skeletal muscle. *Mol Cell Biol* **16**: 4014–4023.

Sambrook J, Fritsch EF, Maniatis T. 1989. *Molecular Cloning. A Laboratory Manual*, 2nd edn. Cold Spring Harbour Laboratory Press: New York.

Samson F, de Jong PJ, Trask BJ, *et al.* 1992. Assignment of the human slow skeletal troponin T gene to 19q13.4 using somatic cell hybrids and fluorescence *in situ* hybridization analysis. *Genomics* **13**: 1374–1375.

Sassaman DM, Dombroski BA, Moran JV, *et al.* 1997. Many human L1 elements are capable of retrotransposition. *Nature Genet* **16**: 37–43.

Schaertl S, Lehrer SS, Geeves MA. 1995. Separation and characterization of the two functional regions of troponin involved in muscle thin filament regulation. *Biochemistry* **34**: 15 890–15 894.

Senapathy P, Shapiro MB, Harris NL. 1990. Splice junctions, branch point sites, and exons: sequence statistics, identification and applications to genome project. *Methods Enzymol* **183**: 252–278.

Copyright © 2003 John Wiley & Sons, Ltd. 

Sharp PA. 1981. Speculations on RNA splicing. *Cell* **23**: 643–646.

Smit AF. 1996. The origin of interspersed repeats in the human genome. *Curr Opin Genet Dev* **6**: 743–748.

Stefancsik R, Jha PK, Sarkar S. 1998. Identification and mutagenesis of a highly conserved domain in troponin T responsible for troponin I binding: potential role for coiled-coil interaction. *Proc Natl Acad Sci USA* **95**: 957–962.

Stefancsik R. 1999. PhD thesis, Sackler Graduate School of Biomedical Sciences, Tufts University, Boston, MA, USA.

Thierfelder L, Watkins H, MacRae C, *et al*. 1994. $\alpha$-Tropomyosin and cardiac troponin T mutations cause familial hypertrophic cardiomyopathy: a disease of the sarcomere. *Cell* **77**: 701–712.

Tobacman LS, Lee R. 1987. Isolation and functional comparison of bovine cardiac troponin T isoforms. *J Biol Chem* **262**: 4059–4064.

Walsh CP, Bestor TH. 1999. Cytosine methylation and mammalian development. *Genes Dev* **13**: 26–34.

Watkins H, McKenna WJ, Thierfelder L, *et al*. 1995. Mutations in the genes for cardiac troponin T and $\alpha$-tropomyosin in hypertrophic cardiomyopathy. *N Engl J Med* **332**: 1058–1064.

Wu QL, Jha PK, Raychowdhury MK, *et al*. 1994. Isolation and characterization of human fast skeletal $\beta$-troponin T cDNA: comparative sequence analysis of isoforms and insight into the evolution of members of a multigene family. *DNA Cell Biol* **13**: 217–233.

Yoder JA, Walsh CP, Bestor TH. 1997. Cytosine methylation and the ecology of intragenomic parasites. *Trends Genet* **13**: 335–340.

Yuan L, Qian N, Tycko B. 1996. An extended region of biallelic gene expression and rodent–human synteny downstream of the imprinted H19 gene on chromosome 11p15.5. *Hum Mol Genet* **5**: 1931–1937.

Copyright © 2003 John Wiley & Sons, Ltd.
